# Cardiac response to chronic restraint stress involves mineralocorticoid receptors in male Sprague–Dawley rats

**DOI:** 10.14814/phy2.70549

**Published:** 2025-10-09

**Authors:** D. Sztechman, T. Zera, K. Czarzasta, E. Szczepanska‐Sadowska, A. Cudnoch‐Jedrzejewska

**Affiliations:** ^1^ Laboratory of Centre for Preclinical Research, Department of Experimental and Clinical Physiology Medical University of Warsaw Warsaw Poland

**Keywords:** echocardiography, eplerenone, glucocorticoid receptors, mineralocorticoid receptors, stress

## Abstract

In the present study, we aimed at elucidating whether exposure to chronic stress affects the expression of cardiac mineralocorticoid receptors (MRs), whether it influences cardiac structure and function, and whether MRs blockade attenuates the stress‐induced changes. Young adult male Sprague–Dawley rats were divided into four groups: rats exposed to restraint stress, rats assigned to eplerenone treatment, rats exposed to restraint stress and eplerenone treatment, and a control group. After 4 weeks of the experiment, rats' blood pressure and heart rate were recorded, transthoracic echocardiography was performed, and blood samples and hearts were collected for further analysis. The study showed that chronic stress increases cardiac glucocorticoid mRNA expression but does not change the expression of MR mRNA. Chronic stress increases matrix metalloproteinases activity in the cardiac muscle. Mild left ventricular pro‐hypertrophic changes were noted on echocardiogram. The changes were partially prevented by MR blockade with the use of eplerenone. These findings indicate that MRs participate in cardiac responses to chronic stress; however, this is not directly associated with significant changes in cardiac MRs expression.

## 1 INTRODUCTION

Glucocorticoids (GCs) and mineralocorticoids are involved in the regulation and remodeling of the cardiovascular system (Oakley & Cidlowski, 2015; Sztechman et al., 2018). Under physiological conditions, GCs circulate in blood at 2–3 orders of magnitude higher concentrations than aldosterone and bind to both glucocorticoid receptors (GRs) and mineralocorticoid receptors (MRs) (Gomez‐Sanchez, 2016; Oakley & Cidlowski, 2015; Sztechman et al., 2018). Expression of GRs and MRs was found in the heart atrial and ventricular tissue (Liu et al., 2019; Sztechman et al., 2018). Transformation of GCs into inactive keto‐analogs in the heart is limited due to low expression of cardiac 11β‐hydroxysteroid dehydrogenase type 2 (11β‐HSD2) (Gomez‐Sanchez & Gomez‐Sanchez, 2021; Sztechman et al., 2018). Thus, physiologically both cardiac MRs and GRs are predominantly occupied by GCs (Herman et al., 2016; Liu et al., 2019).

A response to chronic stress involves behavioral, autonomic, and hormonal adaptations with activation of the hypothalamic–pituitary–adrenal (HPA) axis and release of GCs (Herman et al., 2016). Moreover, increased expression of 11β‐hydroxysteroid dehydrogenase type 1 (11β‐HSD1) in the left ventricle enhances in situ synthesis of GCs and their effects on the MRs and GRs (Matsuura et al., 2015; Nagasawa et al., 2016). Glucocorticoid‐dependent activation of GRs promotes cardiomyocyte hypertrophy and fibrosis; however, it is known that the cardiotoxic effect of GCs is also associated with activation of MRs (Yang et al., 2022).

The crucial role of MRs in the development of cardiovascular diseases is well established and the cardioprotective effect of eplerenone, a selective steroid antagonist of MRs, has been translated into the cardiovascular pharmacotherapy (Fraccarollo et al., 2011; Pitt et al., 1999, 2001; Yang et al., 2022). Despite a relatively short half‐life, the steady‐state eplerenone concentration is reached after repeated or continuous administration (Cook et al., 2003; Kolkhof & Borden, 2012). Therefore, eplerenone is frequently used in studies analyzing the involvement of MRs in the development of cardiovascular diseases.

Thus far, the role of MRs and GRs activation in the pathogenesis of stress‐induced cardiac remodeling and insufficiency has remained unclear. Therefore, in this study, we aimed at elucidating how exposure to chronic stress with a repeated restraint paradigm affects cardiac structure and function, and whether this is associated with alterations in the expression of MRs and GRs in the heart. Furthermore, the effects of MRs blockade by eplerenone on these parameters were evaluated.

## 2 MATERIALS AND METHODS

### 2.1 Animals

All experimental procedures were approved by the local ethical committee on animal experiments in Warsaw. The experiments were carried out in accordance with the Directive 2010/63/EU of the European Parliament and of the Council of the European Union of 22 September 2010 on the protection of animals used for scientific purposes. Experimental procedures were performed on 32 nine‐week‐old male Sprague–Dawley rats weighing 250–280 g at the beginning of the procedure. The rats were housed individually in conventional cages (Euro standard type IV, 1354 G; Tecniplast, Italy) under standardized conditions with a reversed 12‐h dark/12‐h light cycle in a temperature of 20–24°C and air humidity 50 ± 5%. They were fed standard laboratory rodent pellet chow (3.6% fat, 17.4% protein, 60% carbohydrates, 0.2% sodium, and 7.4% crude ash; 286 kcal/100 g; Labofeed B; Kcynia, Poland) and water ad libitum.

### 2.2 Study protocol

**FIGURE 1:**
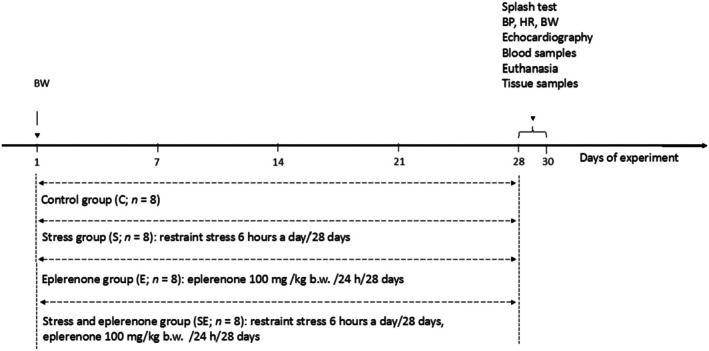
The study protocol. BP, blood pressure; BW, body weight; HR, heart rate.

The 4‐week long experiment was performed on young adult male Sprague–Dawley rats. On the first day of the experiment, body weight was measured in all individuals, and the rats were then divided into the following groups: control group (C; *n* = 8) with non‐restrained untreated individuals; stress group (S; *n* = 8) with untreated animals exposed to chronic restraint stress; eplerenone group (E; *n* = 8) consisting of non‐restrained rats receiving eplerenone; stress and eplerenone group (SE; *n* = 8) with rodents receiving eplerenone and exposed to chronic restraint stress. Eplerenone (Inspra, Pfizer, LOT# Z539803) was administered in drinking water for 28 consecutive days. The dosage of eplerenone (100 mg/kg body weight (b.w.)/24 h) was adjusted to the weekly obtained body weight of each rat and was based on previously published data (Cook et al., 2003; Gromotowicz‐Poplawska et al., 2019). The repeated restraint stress model used in the experiment lasted 6 h/24 h for 28 days and was based on the protocol developed by Magarinos and McEwen (1995). On day 29, all the rats were weighed, a splash test (supportive behavioral marker in the assessment of the stress response) was performed, and heart rate (HR) and blood pressure (BP) were noninvasively recorded with the tail‐cuff volumetric method. On day 30, the transthoracic echocardiography (TTE) was performed, venous blood samples were collected, rats were euthanized, and their hearts were taken for further analysis. All experimental procedures were carried out between 9 a.m. and 4 p.m. in a reversed dark–light cycle matching an active phase of the rats' circadian rhythm. Figure 1 summarizes the study protocol.

### 2.3 Restraint stress

The rats were restrained following the protocol developed by Magarinos and McEwen (1995). The animals were immobilized in restrainers (plexiglass tubes: inner diameter—5.7 cm; length—18 cm; Kent Scientific, Torrington, CT, USA) for 6 h a day for 28 consecutive days. Rats in the restrainers were placed in home cages. During the immobilization, the rats were not able to freely move and were deprived of access to food and water. At the same time, the rats belonging to the non‐stressed groups remained in their home cages and had free access to water and chow.

### 2.4 Splash test

Splash test was performed in all animals to evaluate behavioral response to the immobilization‐induced stress and was performed as described previously (Czarzasta et al., 2019, 2022; Frisbee et al., 2015). Rats were sprayed with 10% sucrose solution. The self‐grooming behavior was recorded for 5 min in a quiet room with a digital Panasonic Lumix DMC‐LX7 camera (Panasonic, Japan). The latency to the first licking (grooming latency) and total time spent on washing both sides and head (grooming time) was analyzed.

### 2.5 Blood pressure and heart rate measurements

Systolic blood pressure (SBP), diastolic blood pressure (DBP) and HR were measured in each rat by the non‐invasive volumetric tail‐cuff method (CODA, Kent Scientific, Torrington, CT, USA) following the American Heart Association recommendations for BP measurements in laboratory animals, as described in detail previously (Kurtz et al., 2005; Segiet et al., 2019). In brief, during BP measurements, each rat stayed in a restrainer placed on a heating plate with the body temperature maintained at 34–35°C as detected at the base of the tail. Then, the cuff was placed on the rat's tail; SBP, DBP, and HR in the tail artery were recorded. In each session, the rats were subjected to two cycles of 10 consecutive BP measurements; then, all technically invalid recordings were rejected. SBP, DBP, and HR used for further statistical analysis were averaged from at least five valid measurements.

### 2.6 Transthoracic echocardiography

Transthoracic echocardiography (TTE) was performed with a Vivid i ultrasound machine (G.E. HealthCare Technologies Inc., USA) geared with an 11 MHz phased‐array probe (10S‐RS, G.E. HealthCare Technologies Inc., USA). Acquisition was carried out at 9–10 MHz, with a frame rate in the range of 200–250 Hz. TTE was performed on rats anesthetized with ketamine (75 mg/kg b.w.; Vetoquinol Biowet, Poland) and xylazine (7 mg/kg b.w.; Vetoquinol Biowet, Poland) given intraperitoneally (i.p.) (Sano et al., 2016). While under anesthesia, in some rats, the small saphenous vein was cannulated with a neonatal catheter (0.62 × 19 G26 KD‐FIX; KD Medical GmbH Hospital Products, Germany) for the administration of the ultrasound enhancing agent (UEA; 4.5 μg/0.1 mL i.v., SonoVue, Bracco International B.V., The Netherlands), as previously described, for improved detection of the endocardial border of the left ventricle in A4C view (Sztechman et al., 2020). After shaving the chest hair, each rat was placed on its back on a heating plate to keep its body temperature stable. During TTE, left ventricular (LV) morphology and function were assessed from the parasternal long (LAX) and short (SAX) axis views and from the apical 4‐ and 5‐chamber views (A4C and A5C). The assessments of the LV end‐diastolic dimension (LVIDd) and the thicknesses of the interventricular septum at diastole (IVSd) as well as LV posterior wall at diastole (LVPWd) were performed from the SAX view using 2D imaging. Fractional shortening (FS) was assessed from 2D‐guided M‐mode imaging from the SAX view (at the level of the papillary muscles). Ejection fraction (EF) was calculated based on LV volumetric measurements with the single‐plane modified Simpson's method. Stroke volume (SV) was calculated based on the velocity time integral (VTI) measurement at the aortic annulus from the apical 5‐chamber view multiplied by aortic annulus diameter measured from the LAX view. Cardiac output (CO) was obtained by multiplying SV by HR. Mitral inflow was recorded by pulsed‐wave Doppler from A4C. The early diastolic mitral annular velocity was assessed with tissue Doppler imaging (TDI) from the A4C view. The A5C view was used for measuring the isovolumic relaxation time (IVRT). All measurements were obtained from three consecutive cardiac cycles and averaged for further statistical analysis. The detailed protocol of echocardiographic examination was published by us previously and followed the guidelines on TTE measurements in experimental animals (Sztechman et al., 2020; Zacchigna et al., 2021). Ventricular dimensions and CO were normalized to the body surface area (BSA) along current recommendations of the European Society of Cardiology for laboratory rodents' echocardiography (Zacchigna et al., 2021). Projections and measurements used for obtaining echocardiographic parameters are presented in Supporting Information (Figure S1).

### 2.7 Blood and cardiac tissue collection

After TTE, the rats received an additional dose of ketamine (100 mg/kg b.w., i.p.). Under deep anesthesia, the right ventricle was cannulated through the fourth intercostal space with a 20‐gauge needle, and a 4 mL venous blood sample was collected in test vials with EDTA anticoagulant. The test tubes were centrifuged for 15 min. at 1702 rcf in 4°C. The plasma was collected into cryovials and stored at −80°C for further analyses. After blood collection, the rats were euthanised by i.p. administration of pentobarbital (Morbital; Biowet Pulawy, Poland) at a dose of 1.5–2 mL/kg b.w. (160 mg/mL of pentobarbital). After euthanasia, the hearts were excised, tissue samples were frozen in liquid nitrogen, and stored at −80°C for further analysis.

#### 2.7.1 Plasma corticosterone, aldosterone, and copeptin analysis (ELISA)

Corticosterone, aldosterone, and copeptin plasma concentrations were measured using enzyme‐linked immunosorbent assay (ELISA) (Corticosterone ELISA Kit: #DEV9922, Demeditec Diagnostics GmbH, Germany; Aldosterone ELISA Kit: #ab136933, Abcam, USA; Rat Vasopressin‐neurophysin 2‐copeptin ELISA Kit: #E0365r, Wuhan EIAab Science Co., Ltd., China). Each ELISA test was carried out in accordance with the instructions provided by the manufacturer.

#### 2.7.2 Analysis of GR mRNA, MR mRNA, and natriuretic peptide B mRNA expression in the left ventricle (RT‐PCR)

Fragments of the left ventricle were homogenized in TRIzol™ Reagent (#15596018, Invitrogen) using Tissue Lyser LT (Qiagen). The total RNA was extracted using a PureLink RNA Mini Kit (#12183018A, Ambion, Life Technologies). A multiplex Real‐Time PCR reaction was carried out in accordance with the Applied Biosystems protocols using the TaqMan® RNA‐to‐Ct™ 1‐Step Kit (#4392938). All primers were obtained from Applied Biosystems. Primers for the target genes were labeled by reporter FAM dye (glucocorticoid receptor: gene symbol Nr3c1, Accession No. Rn00561369_m1; mineralocorticoid receptor: gene symbol Nr3c2, Accession No. Rn00565562_m1; natriuretic peptide B: gene symbol Nppb, Accession No. Rn00580641_m1). Primers for two housekeeping genes were labeled by VIC dye (rat GADPH: gene symbol GAPDH, Accession No. Rn01775763_g1; rat peptidylprolyl isomerase A: gene symbol PPIA, Accession No. Rn00690933_m1). UltraPure™ DNase/RNase‐Free Distilled Water (#10977035, Invitrogen) was used for the RNA template. The amplification reaction was conducted in 40 cycles at 95°C for 15 s and at 60°C for 1 min. The RT‐PCR reactions were performed in a ViiA™ 7 Real‐Time PCR System thermocycler (Applied Biosystems). The relative gene expression was based on comparative quantification to the endogenous controls and given as delta *Ct* (Δ*Ct*) values.

#### 2.7.3 Analysis of MMP‐2, proMMP‐2, and MMP‐9 activity in the left ventricle (gelatin zymography)

Fragments of the left ventricle were homogenized in 500 μL of homogenization buffer (50 mM Tris–HCl, pH 7.6; 150 mM NaCl; 5 mM CaCl_2_; 0.05% Brij‐35; 0.02% NaN_3_) with 1% Triton X‐100 in TissueLyser LT (Qiagen) for 5 min. Samples were then centrifuged for 5 min (12,000 rpm, 4°C) and the supernatant was obtained. Protein concentration was determined in all samples using the SmartSpec™ Plus spectrophotometer (Bio‐Rad) by the Bradford method.

Gelatin zymography was used to detect the activity of MMP‐2 and MMP‐9 in tissue extracts from the left ventricle. Each sample was analyzed in triplicate. Protein separation was performed on 7.5% polyacrylamide gels containing 10% sodium dodecyl sulfate (SDS) and 1 mg/mL of porcine gelatin (Sigma‐Aldrich) as substrate for MMP‐2 and MMP‐9. Samples mixed (1:1) with sample buffer (with 4% SDS) were loaded onto a gel. Either the Recombinant Human MMP‐2 Western Blot Standard Protein (#WBC025, R&D Systems) or Recombinant Human MMP‐9 Western Blot Standard Protein (#WBC018, R&D Systems) was also loaded onto each gel, depending on the type of MMP being tested. The height of which was verified by the molecular weight marker (Precision Plus Protein™ Dual Xtra Prestained Protein Standards, #1610377; Bio‐Rad). After electrophoresis, gels were washed in distilled water and then in a 2.5% solution of Triton X‐100 twice for 20 min to remove SDS. Subsequently, gels were incubated in activation buffer (50 mM Tris–HCl pH 7.6; 200 mM NaCl; 5 mM CaCl_2_; 0.02% NaN_3_; and 1% Triton X‐100) at 37°C for 18 h. Gels were then stained in 0.1% Coomassie Brilliant Blue R250 in 40% methanol and 10% acetic acid solution for 1 h and destained in 40% methanol and 10% acetic acid to obtain clear bands against a blue background. The above process caused the disappearance of the molecular weight ladder. The zymograms were scanned and quantified by densitometry using a ChemiDoc Imaging System (ChemiDoc™ MP System, Bio‐Rad). Proteolytic MMP‐2 and MMP‐9 activity was determined in arbitrary units in comparison with MMP‐2 and MMP‐9 standards, respectively.

### 2.8 Statistics

**FIGURE 2:**
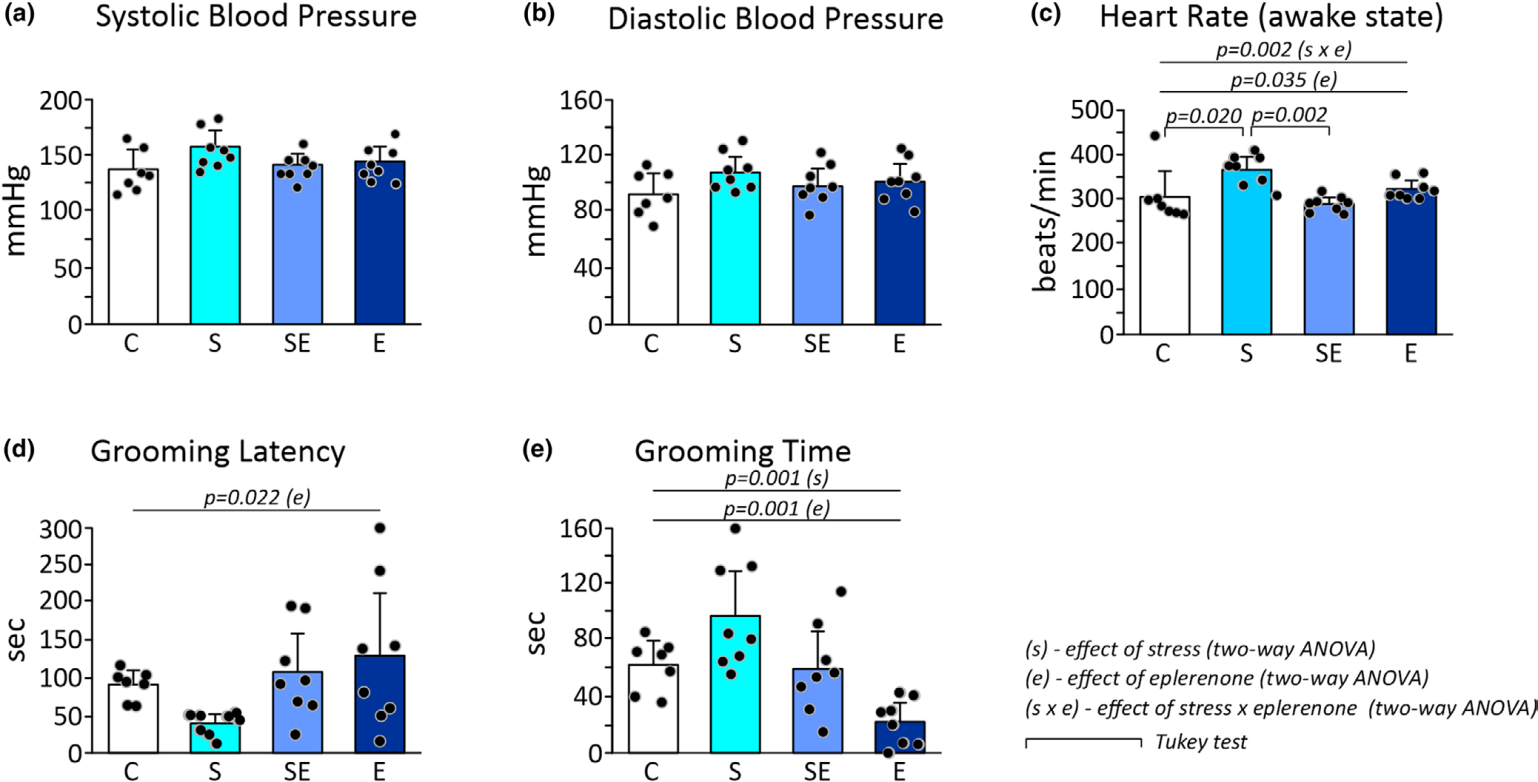
Effects of chronic restraint stress and eplerenone treatment on blood pressure and heart rate in awake rats. (a) Systolic blood pressure. (b) Diastolic blood pressure. (c) Heart rate. Effects of chronic stress and eplerenone treatment on behavioral reactions in splash test. (d) Latency to the first grooming. (e) Total grooming time. C, control group; E, eplerenone group; S, stress group; SE, stress and eplerenone group. Dots correspond to individual measurements.

**FIGURE 3:**
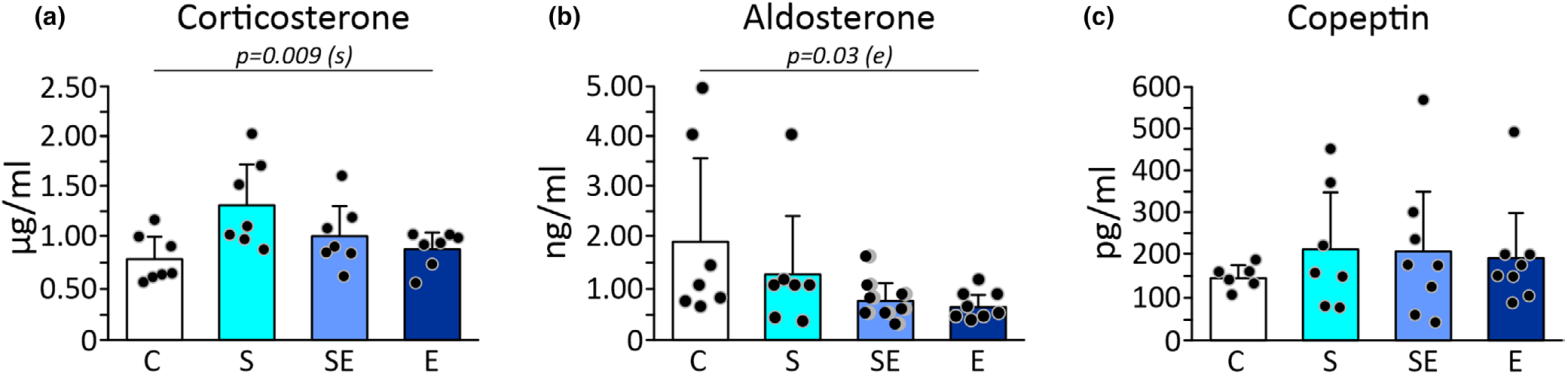
Effects of chronic restraint stress and eplerenone treatment on concentrations of plasma hormones. (a) Plasma corticosterone concentration. (b) Plasma aldosterone concentration. (c) Plasma copeptin concentration. C, control group; E, eplerenone group; S, stress group; SE, stress and eplerenone group. Dots correspond to individual measurements.

**FIGURE 4:**
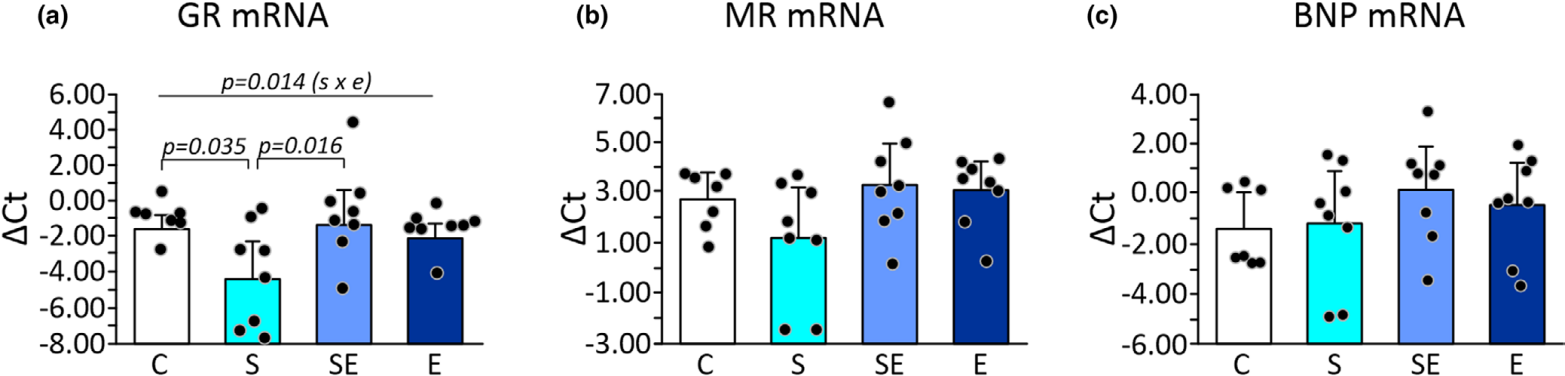
Effects of chronic restraint stress and eplerenone treatment on cardiac GR mRNA, MR mRNA, and BNP mRNA expression in the left ventricle. (a) Glucocorticoid receptor mRNA expression. (b) Mineralocorticoid receptor mRNA expression. (c) Brain natriuretic peptide mRNA expression. C, control group; E, eplerenone group; S, stress group; SE, stress and eplerenone group. Dots correspond to individual measurements.

**FIGURE 5:**
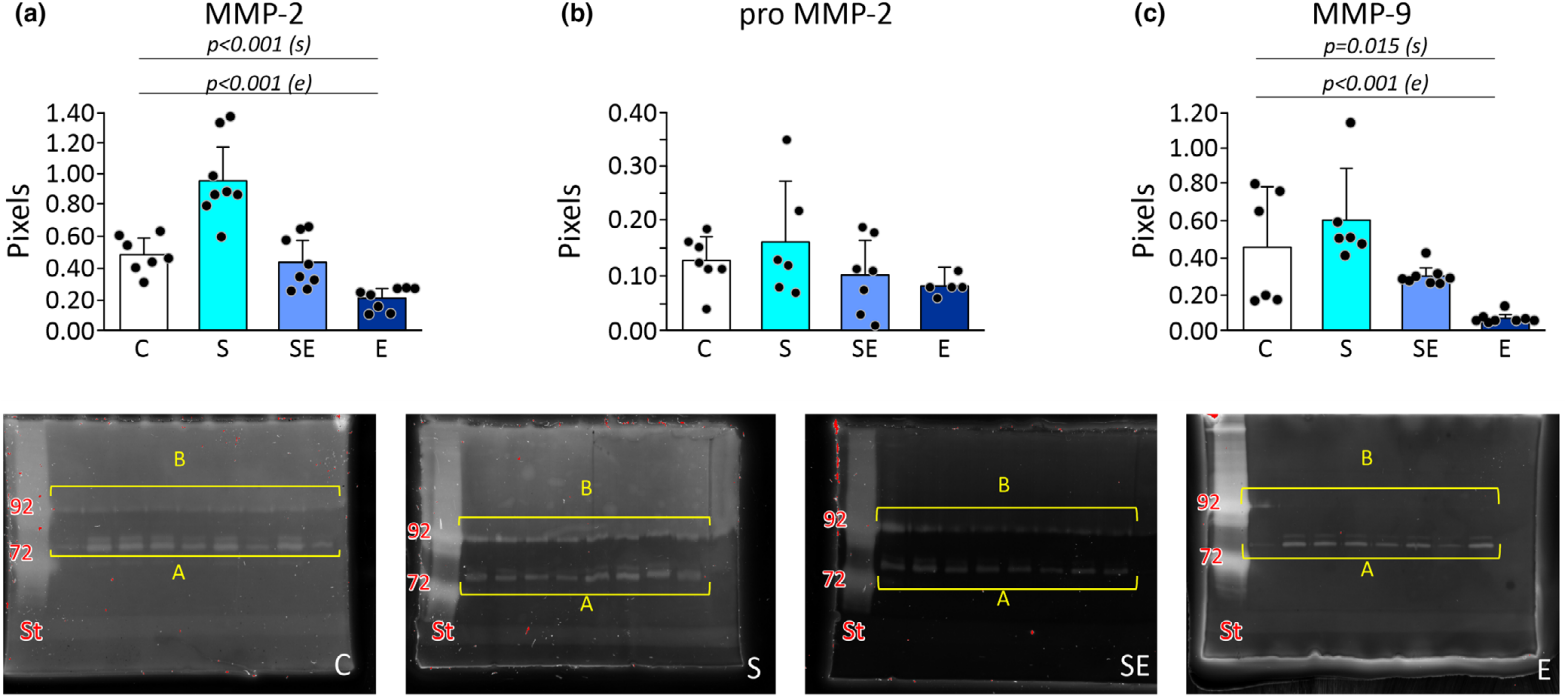
Effects of chronic restraint stress and eplerenone treatment on matrix metalloproteinases' activity (zymography) in the left ventricle. Top row: (a) Matrix metalloproteinase‐2 activity. (b) Pro‐matrix metalloproteinase‐2 activity. (c) Matrix metalloproteinase‐9 activity. C, control group; E, eplerenone group; S, stress group; SE, stress and eplerenone group. Dots correspond to individual measurements. Lower row: Representative gelatin zymograms showing gelatinolytic activities of MMP‐9. Rats' left ventricular homogenate samples were obtained from the control group (C), the stress group (S), the stress and eplerenone group (SE), and the eplerenone group (E). Gelatinolytic bands of MMP‐9 were detected using a molecular weight (MW) marker and MMP‐9 protein standard. Lower bands on MMP‐9 gels, corresponding to ~72 kDa, were identified as active MMP‐2 based on their molecular weight, confirmed using gels containing an MW ladder and MMP‐2 protein standard. A: Gelatinolytic activities of MMP‐9 (~92 kDa). B: Gelatinolytic bands of active MMP‐2 (~72 kDa). Analysis was performed in triplicate. St‐ protein standard. The wide lysis zone indicates enzymatic activity of the MMP‐9 protein standard used or nonspecific gelatinolysis. The additional band corresponding to ~72 kDa likely originates from biological samples.

**FIGURE 6:**
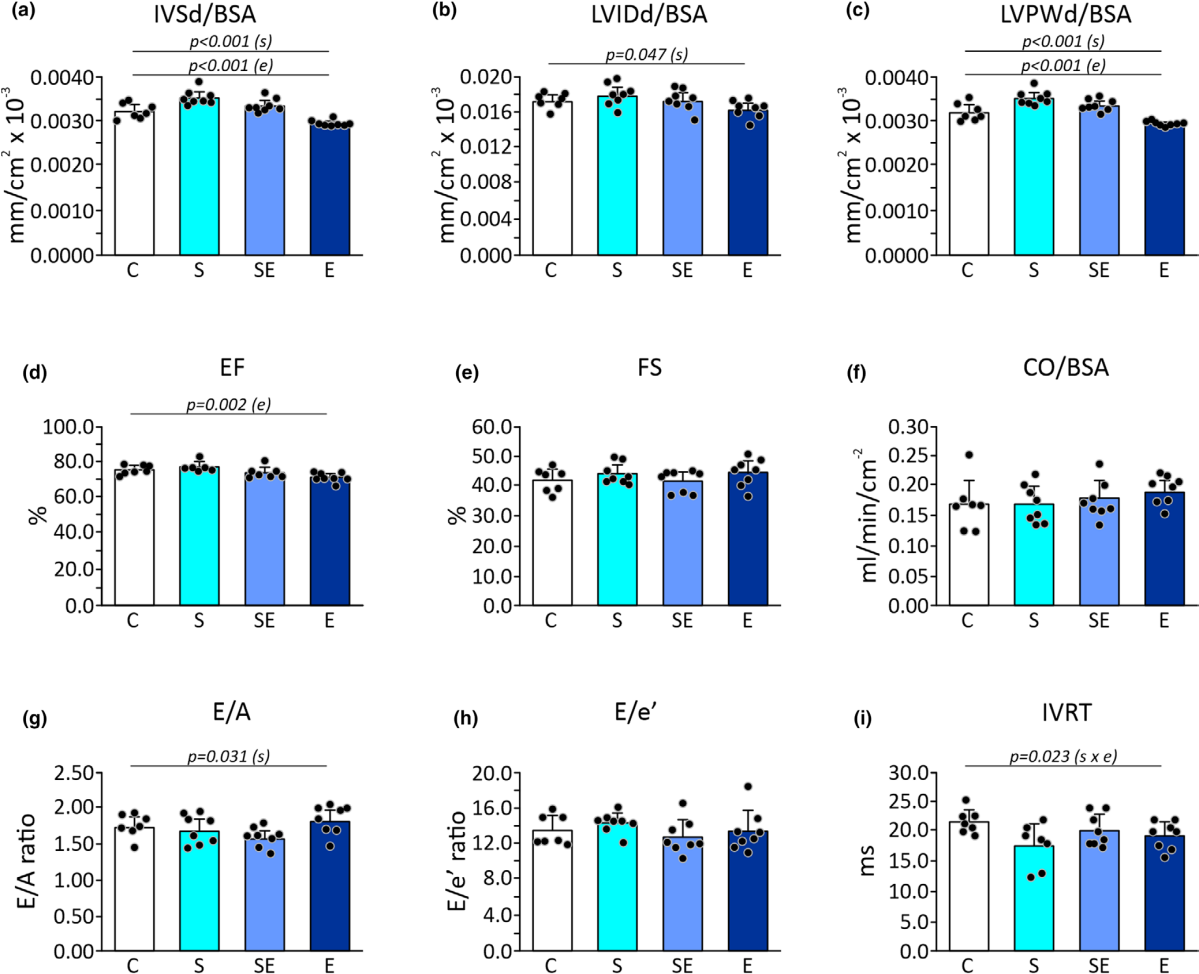
The effect of chronic stress and eplerenone‐treatment on echocardiographic parameters. (a) Interventricular septum at diastole indexed to BSA (IVSd/BSA). (b) LV internal dimension at diastole indexed to BSA (LVIDd/BSA). (c) LV posterior wall at diastole indexed to BSA (LVPWd/BSA). (d) Ejection fraction (EF). (e) Fractional shortening (FS). (f) Cardiac output indexed to BSA (CO/BSA). (g) Early to late diastolic transmitral flow velocity (E/A). (h) Early diastolic transmitral flow velocity to early diastolic mitral annular velocity (E/e′). (i) Isovolumic relaxation time (IVRT). C, control group; E, eplerenone group; S, stress group; SE, stress and eplerenone group. Dots correspond to individual measurements.

A two‐way analysis of variance (ANOVA) was used to investigate whether chronic stress, eplerenone treatment, or the simultaneous effect of both factors had a modifying effect on the measured parameters. When statistically significant results were obtained in the two‐way ANOVA for the interaction effect of stress and eplerenone, a Tukey post hoc test was performed. The statistically significant differences were considered if *p* < 0.05. Data are expressed as means with standard deviation (SD). Bar charts show means ± SD for variables for C, S, SE, and E groups. Statistical analysis was performed with the use of Statistica software version 13.3 (TIBCO Software Inc., Palo Alto, CA, USA). Results of ANOVA and Tukey post hoc test are shown in Figures 2, 3, 4, 5, 6, Table S1 (ANOVA), and Table S2 (Tukey test). Mean values of variables obtained in each group are shown in Figures 2, 3, 4, 5, 6 and Tables S3–S7.

## 3 RESULTS

### 3.1 The effect of stress, eplerenone, and interactions of stress and eplerenone on SBP, DBP, and HR

Two‐way ANOVA did not show a statistically significant effect of stress, eplerenone treatment, as well as stress and eplerenone interaction on SBP (Figure 2a; Table S1) and DBP (Figure 2b; Table S1). However, there was a statistically significant effect of eplerenone treatment (*p* = 0.035) and a significant effect of the interaction of stress and eplerenone (*p* = 0.002) on HR recorded with tail‐cuff in the awake state (Figure 2c; Table S1). The Tukey post hoc analysis showed significantly higher HR in the S group in comparison to the C group (*p* = 0.02) and the SE group (*p* = 0.002) (Figure 2c; Tables S2 and S3). Two‐way ANOVA for HR measured during TTE under anesthesia was insignificant for the effect of stress or eplerenone (Table S1).

Mean values of SBP, DBP, and HR obtained in each group are presented in Figure 2 (upper part) and Table S3.

### 3.2 The effect of chronic stress, eplerenone, and interactions of stress and eplerenone on grooming latency and grooming time (Splash test)

Two‐way ANOVA showed a statistically significant effect of eplerenone (*p* = 0.022) on grooming latency, with the latency longer in the eplerenone‐treated groups in comparison with non‐treated ones (Figure 2d; Tables S1 and S3). There was no significant effect of stress nor interaction of stress and eplerenone on grooming latency (Figure 2d; Table S1).

Two‐way ANOVA showed significant effects of stress (*p* = 0.001) and eplerenone treatment (*p* = 0.001) on grooming time, which was longer in the stressed groups in comparison with non‐stressed groups and shorter in eplerenone‐treated groups in comparison with non‐treated ones, respectively (Figure 2e; Tables S1 and S3). There was no significant interaction effect of stress and eplerenone on grooming time (Figure 2e; Table S1).

Mean values of grooming latency and grooming time obtained in each group are presented in Figure 2 (lower part) and Table S3.

### 3.3 The effect of chronic stress, eplerenone, and the interaction of stress and eplerenone on body weight change

Two‐way ANOVA showed a statistically significant effect of stress (*p* < 0.001) on body weight change from baseline (Table S1). In stressed groups, weight loss was found in comparison with non‐stressed groups (Table S3). The analysis showed an insignificant effect of eplerenone treatment and an insignificant effect of eplerenone‐stress interaction on body weight change from baseline (Table S1).

### 3.4 The effect of chronic stress, eplerenone, and interaction of stress and eplerenone on plasma corticosterone, aldosterone, and copeptin concentration

Two‐way ANOVA showed a statistically significant effect of stress (*p* = 0.009) on plasma corticosterone concentration (Figure 3a; Table S1). The stressed groups had a higher plasma corticosterone level compared to the unstressed animals (Figure 3a; Table S4). The analysis showed no statistically significant effect of eplerenone or the interaction of stress and eplerenone on plasma corticosterone concentration (Figure 3a; Table S1).

Two‐way ANOVA showed a significant effect of eplerenone (*p* = 0.03) on plasma aldosterone concentrations (Figure 3b; Table S1). In eplerenone‐treated groups, lower plasma aldosterone levels were noted in comparison with untreated animals (Figure 3b; Table S4). Two‐way ANOVA showed a statistically insignificant effect of stress as well as stress and eplerenone interaction on plasma aldosterone concentration (Figure 3b; Table S1).

Two‐way ANOVA showed the absence of a statistically significant effect of stress, eplerenone as well as stress and eplerenone interaction on plasma copeptin concentration (Figure 3c; Table S1).

Mean values of plasma corticosterone, aldosterone, and copeptin concentrations obtained in each group are presented in Figure 3 and Table S4.

### 3.5 The effect of chronic stress, eplerenone, and the interaction of stress and eplerenone on the expression of GR mRNA, MR mRNA, and BNP mRNA in the left ventricle

Two‐way ANOVA showed a statistically significant effect of the stress and eplerenone interaction (*p* = 0.014) on the GR mRNA expression in the left ventricle (Figure 4a; Table S1). The Tukey post hoc analysis showed statistically significant differences between the S group and the C group (*p* = 0.035; Figure 4a; Table S2). The S group had a significantly higher GR mRNA expression compared to the C group (Figure 4a; Table S5). The Tukey post hoc test also showed statistically significant differences between the S group and the SE group (*p* = 0.016; Figure 4a; Table S2). The S group had a significantly higher GR mRNA expression compared to the SE group (Figure 4a; Table S5).

Two‐way ANOVA did not show a statistically significant effect of stress, eplerenone, as well as the interaction of stress and eplerenone on the expression of MR mRNA in the left ventricle (Figure 4b; Table S1).

Two‐way ANOVA did not show a statistically significant effect of stress, eplerenone, and an interaction of stress and eplerenone on the expression of BNP mRNA in the left ventricle (Figure 4c; Table S1).

All mean values of mRNA expression are presented in Figure 4 and Table S5.

### 3.6 The effect of chronic stress, eplerenone, and interaction of stress and eplerenone on matrix metalloproteinases activity in the left ventricle

Two‐way ANOVA showed a statistically significant effect of stress (*p* < 0.001) on MMP‐2 activity in the left ventricle (Figure 5a; Table S1). In stressed groups, an increase in MMP‐2 was recorded in comparison to non‐stressed animals (Figure 5a; Table S6). Two‐way ANOVA showed a significant effect of eplerenone (*p* < 0.001) on MMP‐2 activity in the left ventricle (Figure 5a; Table S1). In eplerenone‐treated rats, a decrease in MMP‐2 activity was found in comparison with untreated groups (Figure 5a; Table S6). Two‐way ANOVA did not reveal a statistically significant effect of stress and eplerenone interaction on MMP‐2 activity in the left ventricle (Figure 5a; Table S1).

Two‐way ANOVA did not show a statistically significant effect of stress, eplerenone, and the interaction of stress and eplerenone on pro‐MMP‐2 activity (Figure 5b; Table S1).

Two‐way ANOVA showed a significant effect of stress (*p* = 0.015) on MMP‐9 activity in the left ventricle (Figure 5c; Table S1). In stressed rats, an increase in MMP‐9 activity was noted in comparison to non‐stressed animals (Figure 5c; Table S6). Two‐way ANOVA also showed a statistically significant effect of eplerenone (*p* < 0.001) on MMP‐9 activity in the left ventricle (Figure 5c; Table S1). In eplerenone‐treated rats, a decrease in MMP‐9 activity was recorded in comparison with untreated animals (Figure 5c; Table S6). However, two‐way ANOVA did not show a significant effect of the stress and eplerenone interaction on MMP‐9 activity in the left ventricle (Figure 5c; Table S1). Mean values of matrix metalloproteinases' activity obtained in each group are presented in Figure 5 and Table S6.

### 3.7 The effect of chronic stress, eplerenone, and interaction of stress and eplerenone on echocardiographic parameters

#### 3.7.1 LVIDd, and the thicknesses of the IVS and LVPW at diastole

Two‐way ANOVA showed a statistically significant effect of stress (*p* < 0.001) on the thickness of IVSd/BSA (Figure 6a; Table S1). In rats exposed to stress, increased IVSd/BSA thickness was found when compared to non‐stressed animals (Figure 6a; Table S7). Two‐way ANOVA showed a statistically significant effect of eplerenone (*p* < 0.001) on the thickness of IVSd/BSA (Figure 6a; Table S1). In rats receiving eplerenone, decreased IVSd/BSA thickness was found in comparison with untreated animals (Figure 6a; Table S7). However, two‐way ANOVA did not show a statistically significant effect of stress and eplerenone interaction on IVSd/BSA (Figure 6a; Table S1).

Two‐way ANOVA showed a statistically significant effect of stress (*p* = 0.047) on LVIDd/BSA (Figure 6b; Table S1). In stressed rats, an increase in LVIDd/BSA was recorded in comparison with non‐stressed animals (Figure 6b; Table S7). However, two‐way ANOVA did not show a significant effect of eplerenone as well as stress and eplerenone on LVIDd/BSA (Figure 6b; Table S1).

Two‐way ANOVA showed a statistically significant effect of stress (*p* < 0.001) on LVPWd/BSA (Figure 6c; Table S1). In stressed rats, higher values of LVPWd/BSA were recorded when compared to unstressed animals (Figure 6c; Table S7). Two‐way ANOVA showed a statistically significant effect of eplerenone (*p* < 0.001) on LVPWd/BSA (Figure 6c; Table S1). In eplerenone‐treated rats, lower LVPWd/BSA thickness was found when compared to the thickness of LVPWd/BSA in untreated animals (Figure 6c; Table S7). Two‐way ANOVA did not show a significant effect of stress and eplerenone interaction on LVPWd/BSA (Figure 6c; Table S1).

Mean values of IVSd, LVIDd, and LVPWd obtained in each group are presented in Figure 6 and Table S7.

#### 3.7.2 Left ventricular systolic function

Two‐way ANOVA showed a statistically significant effect of eplerenone (*p* = 0.002) on EF (Figure 6d; Table S1). In eplerenone‐treated rats, lower EF was reported in comparison with untreated animals (Figure 6d; Table S7). However, it should be noted that in all groups, the EF value was within physiological norms. Two‐way ANOVA did not show a significant effect of stress as well as the interaction of stress and eplerenone on EF (Figure 6d; Table S1).

Two‐way ANOVA did not show a significant effect of stress, eplerenone, and the interaction of stress and eplerenone on FS (Figure 6e; Table S1).

Two‐way ANOVA did not show a significant effect of stress, eplerenone, and the interaction of stress and eplerenone on stroke volume and cardiac index (cardiac output indexed to BSA) (Figure 6f; Table S1).

Mean values of EF, FS, and CO obtained in each group are presented in Figure 6 and Table S7.

#### 3.7.3 Left ventricular diastolic function

Two‐way ANOVA showed a significant effect of stress (*p* = 0.031) on the E/A ratio (Figure 6g; Table S1). In stressed rats, a lower value of the mitral E/A ratio was reported in comparison with unstressed animals (Figure 6g; Table S7). However, it should be noted that the E/A ratio recorded in both stressed groups is within physiological norms. Two‐way ANOVA did not show a significant effect of eplerenone as well as the interaction of stress and eplerenone on the E/A ratio (Figure 6g; Table S1).

Analysis with the use of two‐way ANOVA did not show a significant effect of stress, eplerenone, and the interaction of stress and eplerenone on the E/e′ ratio (Figure 6h; Table S1).

Two‐way ANOVA did not show significant effect of stress as well as eplerenone on IVRT (Figure 6i; Table S1). The analysis showed significant effect of stress and eplerenone interaction (*p* = 0.023) on IVRT, however Tukey post hoc test did not show statistically significant differences between groups (Figure 6i; Tables S1 and S2).

Mean values of E/A, E/e′, and IVRT obtained in each group are presented in Figure 6 and Table S7.

## 4 DISCUSSION

The results of our study show that exposure to chronic restraint stress increases plasma corticosterone concentration and expression of GR mRNA in the left ventricle, which is associated with increased activity of MMP‐2, MMP‐9, and leads to cardiac remodeling. Chronic stress has an insignificant effect on plasma aldosterone concentration and expression of cardiac MR mRNA; nevertheless, MRs blockade may alter or prevent stress‐induced changes. We discuss these findings in the context of the role of MRs and their blockade in the pathogenesis of cardiovascular disorders during stress.

### 4.1 Increased HR, altered behavior, and change in body weight as supportive markers in the assessment of the developing stress response

Previous studies have shown that repeated restraint stress has an impact on resting HR in rats (Morais‐Silva et al., 2019; Sikora et al., 2016). We obtained similar results while noting that eplerenone reversed these effects. This finding is in line with other studies showing that brain MRs participate in the autonomic regulation of the cardiovascular response to stress, and blockade of MRs in the brainstem reduces the pressor reflex and diminishes the tachycardic response (Chin et al., 2019; Downey et al., 2017; Gomez‐Sanchez, 2016; Kang et al., 2006). We did not record significant differences in SBP and DBP across all groups.

Delayed body weight gain or loss of body weight is typical in chronically stressed rats (Marin et al., 2007). In our study, we observed a decrease in body weight in stressed groups, while there was no effect of eplerenone on body weight.

Assessing the development of stress response, we also noted behavioral alterations (evaluated by the splash test) in chronically restrained rats. Eplerenone reversed these changes, presumably by blocking brain MRs, which was confirmed by other studies (Gomez‐Sanchez, 2016; Hlavacova et al., 2010; Kang et al., 2006).

### 4.2 Chronic restraint stress elevates plasma corticosterone concentration

Elevated plasma GCs level is a hormonal marker of the stress reaction and contributes to cardiovascular complications (Deng et al., 2023; van der Wal et al., 2022). In our study, stressed groups showed elevated concentrations of plasma corticosterone. We also noted a lack of effect of eplerenone on plasma corticosterone level. Our results acknowledge that eplerenone does not interfere directly with GCs synthesis in the adrenals (Ye et al., 2009). Nevertheless, peripherally administered eplerenone can penetrate into the brain; thus, it is likely that the blockade of brain MRs may alleviate activation of the HPA axis and affect adrenal gland function (Gomez‐Sanchez, 2016; Hlavacova et al., 2010; Kang et al., 2006).

It is noteworthy that in states with HPA axis activation, GCs synthesis in the heart increases, which can lead to a local increase of GCs concentration in comparison to plasma (Gray et al., 2017; Taves et al., 2011). The MRs blockade with eplerenone decreases the expression of cardiac 11β‐HSD1, a key enzyme for corticosterone synthesis in the left ventricle (Hori et al., 2017).

Exposure to stress may enhance activity of the vasopressinergic system; however, neither of the groups showed significant changes in plasma copeptin concentration (a surrogate marker of vasopressin release) (Szczepanska‐Sadowska et al., 2010).

### 4.3 Chronic restraint stress and administration of eplerenone do not influence plasma aldosterone concentration

Several studies document an increase in plasma aldosterone concentration (PAC) during acute stress; however, a lack of increase in PAC under long‐term stressful stimuli is also described (Gideon et al., 2020; Lehoux et al., 1998; Ulrich‐Lai et al., 2006). Under chronic stress, increased activity of the zona fasciculata synthesizing GCs with attenuated function of the zona glomerulosa releasing mineralocorticoids was observed in the adrenals, which may explain the lack of changes in PAC among groups found in our study (Ulrich‐Lai et al., 2006). In contrast to previous reports, in the present study, eplerenone did not elicit an increase in PAC. Similarly, some research indicates that PAC elevation after eplerenone treatment is not observed in hypothyroidism or kidney diseases with hyperkalemia (Dizaye & Mustafa, 2019; Shavit et al., 2011). It is known that chronic stress‐induced GCs excess causes thyroid gland suppression, and eplerenone belongs to potassium‐sparing diuretics (Benker et al., 1990; Castillo‐Campos et al., 2021; Gomez‐Sanchez, 2016; Roelfsema et al., 2009). Perhaps an assessment of the function of the thyroid gland, kidneys, and plasma electrolyte composition would provide an additional explanation of our results.

In our study, plasma corticosterone and aldosterone concentrations were higher than reported by other researchers, which was probably caused by the use of anesthesia with a mixture of ketamine and xylazine during blood sampling (Pereira et al., 2021; Radford et al., 2018). Additionally, the ELISA kit used in our study typically shows higher values of corticosterone, as reported previously (Cudnoch‐Jedrzejewska et al., 2015).

### 4.4 Chronic stress and eplerenone affect expression of GR mRNA but not MR mRNA in the left ventricle

Physiologically, HPA axis regulation is based on a negative feedback mechanism, and high GCs concentration decreases the GRs expression both at the mRNA and protein levels (Chiba et al., 2012; Gadek‐Michalska et al., 2013; Harris et al., 2001). However, in pathological states with hypercortisolemia, GR mRNA transcription may also increase (Hori et al., 2024; Nagasawa et al., 2016). In our study, exposure to chronic stress caused an increase in GCs plasma concentration and expression of GR mRNA in the left ventricle. These results allow us to assume that chronic stress‐induced HPA axis dysfunction can be connected rather to decreased sensitivity of GRs than decreased GRs expression (Hartmann et al., 2021; Hori et al., 2024; Kunugi et al., 2006; Lewis‐Tuffin & Cidlowski, 2006). As we observed in our study, MR blockade can attenuate this process.

Nonetheless, we found insignificant changes in cardiac MR mRNA expression in all the groups, which suggests no correlation between GCs concentration and MR expression degree. On the other hand, we found that during chronic stress, eplerenone significantly influenced the biochemical and echocardiographic parameters we evaluated, as discussed below.

### 4.5 Chronic stress increases, whereas eplerenone decreases expression of cardiac MMPs

Our study indicates that chronic stress increases the activity of cardiac MMP‐2 and MMP‐9. The direct inhibitory effect of GCs on MMPs activity (via MRs) and consequently cardiac hypertrophy and fibrosis is well documented (Omori et al., 2014; Xia et al., 2007). However, under chronic stress, MMPs may be activated by angiotensin II and adrenergic stimulation (Roy et al., 2009; Senzaki et al., 2000; Surinkaew et al., 2019; Yin et al., 2023). Prolonged MMPs activation leads to dynamic cardiac extracellular matrix (ECM) turnover, progressive LV dilatation, and heart failure (HF) (Peterson et al., 2001).

A large amount of evidence indicates the important role of MRs in the regulation of the MMPs expression (Chi et al., 2014; Martin‐Fernandez et al., 2011). Association between MRs stimulation and the activity of MMPs may be of particular importance in chronic stress, as the binding of GCs to MRs can mimic aldosterone action and promote cardiac remodeling (Mihailidou et al., 2009; Rickard et al., 2012). Inhibition of MMPs prevents this process and slows the progression of HF (Chancey et al., 2002; Chi et al., 2014; Gajarsa & Kloner, 2011; Miura et al., 2003; Peterson et al., 2001). Our study confirms these observations, demonstrating that eplerenone exerts a protective effect by inhibiting the activity of cardiac MMPs.

### 4.6 Pro‐hypertrophic changes in the left ventricle induced by chronic stress are partially prevented by eplerenone

There is little research evaluating the impact of chronic stress on cardiac muscle in healthy rats (Czarzasta et al., 2024). In stress‐exposed rats, we noted a subtle increase in the thicknesses of IVSd and LVPWd and an increase in LVIDd compared to the control group; nevertheless, echocardiographic parameters in all groups were within physiological values reported for Sprague–Dawley rats (Zacchigna et al., 2021). We associate these changes with GCs' action (via GRs, MRs, and indirectly by adrenergic stimulation) as well as increased LV volume load (compensatory response enabling maintaining proper stroke volume) (Gajarsa & Kloner, 2011; Lister et al., 2006; Miura et al., 2003; Pitoulis & Terracciano, 2020; Roten et al., 2000; Spinale et al., 1998). Nonetheless, we did not find systolic or diastolic dysfunction or significant differences in the level of BNP mRNA expression (a marker of cardiac function) across all groups (McDonagh et al., 2021; Potter et al., 2009).

Our findings are in line with preclinical and clinical studies. Specifically, elevated levels of MMP‐9 were found to be associated with increased LV diastolic dimensions and increased LV wall thickness in patients participating in the Framingham Heart Study (Sundstrom et al., 2004). Cardioprotective effects of MR blockade on cardiac MMPs activity and LV remodeling were also reported (Chancey et al., 2002; Chi et al., 2014; Hori et al., 2017; Peterson et al., 2001).

**FIGURE 7:**
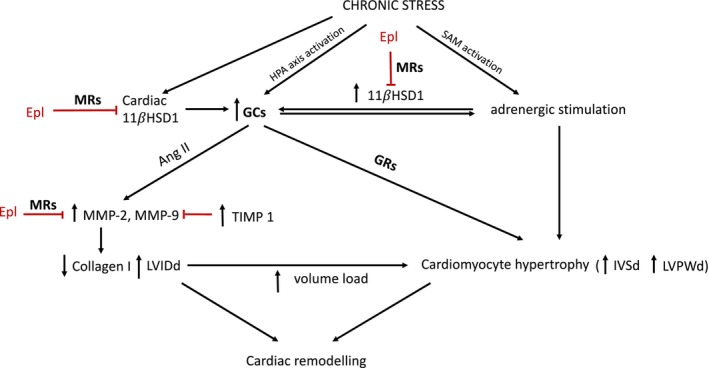
Potential pathomechanisms involved in cardiac response to stress exposure, suggested by the present study and other investigations. The developing stress response results in activation of the HPA axis accompanied by increased synthesis and secretion of GCs (Herman et al., 2003). Parallelly, there is sympathetic adrenomedullary (SAM) activation and increased adrenergic stimulation (Godoy et al., 2018). Increased synthesis and secretion of GCs is also influenced by increased 11β‐HSD1 activity, which is also increased by adrenergic stimulation (Hori et al., 2017; Nagasawa et al., 2016). The stimulatory effect of GCs on adrenergic activity has also been documented (Lister et al., 2006). One effect of β‐adrenergic stimulation and GCs‐induced increase in Ang II activity is an elevation of MMP‐2 activity in cardiac tissue (Gajarsa & Kloner, 2011; Miura et al., 2003; Roy et al., 2009). In response, TIMP‐1 activity increases, but the stoichiometric ratio of MMP‐2 and TIMP activation in favor of MMP‐2 induces an increase in collagen I degradation processes in the ECM (Spinale et al., 2002). At the same time, cardiomyocyte hypertrophy stimulated by excess GCs is observed (Miura et al., 2003; Ren et al., 2012). Increased collagen degradation participates in a dynamic cardiac ECM turnover, which, together with concomitant LV cardiomyocyte hypertrophy, is accompanied by progressive eccentric remodeling with enlargement of the LV cavity, increased volume load, and increased LV muscle thickness (Pitoulis & Terracciano, 2020; Roten et al., 2000; Spinale et al., 1998). By reducing the activity of MMP‐2 and 11β‐HSD1, eplerenone inhibits one of the likely pathomechanisms of chronic stress‐induced LV remodeling (Chancey et al., 2002; Chi et al., 2014; Hori et al., 2017; Peterson et al., 2001). 11β‐HSD1, 11β‐hydroxysteroid dehydrogenase type 1; ECM, extracellular matrix; EPL, eplerenone; GRs, glucocorticoid receptors; HPA axis, hypothalamic–pituitary–adrenal axis; IVSd, interventricular septum at diastole; LVIDd, left ventricular internal diameter at diastole; LVPWd, left ventricular posterior wall at diastole; MMP‐2, matrix metalloproteinase‐2; MMP‐9, matrix metalloproteinase‐9; MRs, mineralocorticoid receptors; SAM, sympathetic adrenomedullary system; TIMP‐1, tissue inhibitor of metalloproteinase‐1.

The potential mechanisms involved in stress‐induced remodeling of the left ventricle and protective actions of eplerenone are summarized in Figure 7.

## 5 LIMITATIONS

Our study does not allow determining several essential issues. Evaluation of the GR and MR expressions at the protein level, GRs and MRs intracellular pathways, as well as the estimation of the expression of β‐HSD isoforms in the cardiac tissue would allow for a better understanding of the cross‐talk between MRs and GRs. The use of selective antagonists of GRs would further delineate the involvement of these receptors in cardiac remodeling. Another limitation is the lack of assessment of glucocorticoid and mineralocorticoid synthesis in heart tissues. The lack of histopathological analysis of the left ventricle for examination of fibrosis and cardiomyocyte geometry does not allow for more thorough insight into the mechanisms of the observed changes. Assessment of other markers such as the atrial natriuretic peptide (ANP) and enzymes involved in the processing of natriuretic peptides would provide a broader picture of molecular changes associated with stress and MRs, especially given the anti‐fibrotic and anti‐hypertrophic effects of natriuretic peptides. In our study, only male rats were investigated, while it is known that the effects of steroid hormones are strongly affected by sex, and future experiments should include both females and males. The other weakness of our study is the lack of procedures for evaluation of the habituation to the restraint stress; however, it should be noted that hemodynamic adaptation was not found in rats exposed to repeated restraint stress, although at a lower intensity than in our study.

## 6 CONCLUSIONS

In conclusion, our results show that exposure to chronic restraint stress in normotensive Sprague–Dawley rats leads to increased plasma corticosterone concentration, increased expression of GR mRNA, and activation of extracellular matrix enzymes, which gives rise to cardiac remodeling. The reported changes depend on the activation of MRs, as they may be prevented or attenuated by the administration of eplerenone, a selective MR antagonist. In this light, mineralocorticoid receptors should be further investigated in the context of their pharmacological potential in the treatment of pathologies associated with exposure to stress.

## AUTHOR CONTRIBUTIONS

D.S. contributed to the design of the study, developed the echocardiographic protocol, performed echocardiography, carried out experiments, analyzed data, prepared figures, wrote the draft and the final version of the manuscript. K.C. performed gelatin zymography, RT‐PCR, ELISA tests, participated in the experiments, analyzed data, and reviewed the final version of the manuscript. E.S.‐S. designed the study and reviewed the final version of the manuscript. A.C.‐J. reviewed the final version of the manuscript. T.Z. contributed to the design of the study, supervised experiments, analyzed results, prepared figures, and contributed to the draft and final versions of the manuscript.

## FUNDING INFORMATION

The study and publication were financed by statutory funding of the Department of Experimental and Clinical Physiology, Medical University of Warsaw.

## CONFLICT OF INTEREST STATEMENT

No conflicts of interest are declared by the authors.

## ETHICS STATEMENT

This study was ethically approved by the Local Ethical Committee on Animal Experiments in Warsaw, Poland (Ethical Committee resolution numbers 621/2018 and WAW2/025/2019).

## Supporting information


Appendix S1.

## Data Availability

Data will be made available on demand.
