# Supplementary material for: Cardiac response to chronic restraint stress involves mineralocorticoid receptors in male Sprague–Dawley rats
Source: Physiol Rep. 2025 Oct 9;13(19):e70549. doi: 10.14814/phy2.70549 (PMC12510903; doi:10.14814/phy2.70549)
Supplement: Supplementary file 1 — Appendix S1. [file PHY2-13-e70549-s001.zip › Table_S1; Table_S2.docx]

**Table S1**. The results of two-way analysis of variance (ANOVA) and p-values for variables evaluated in groups of rats treated with stress alone (Stress), eplerenone alone (Eplerenone) or stress associated with eplerenone (Stress x Eplerenone).

| Variable | Treatment | *MS* | *F* | *df* | *p* | η^2^ |
| --- | --- | --- | --- | --- | --- | --- |
| Systolic blood pressure [mmHg] | Stress | 545.09 | 2.17 | 1; 27 | 0.153 | 0.07 |
|  | Eplerenone | 150.89 | 0.60 | 1; 27 | 0.445 | 0.02 |
|  | Stress x Eplerenone | 988.40 | 3.93 | 1; 27 | 0.058 | 0.13 |
| Diastolic blood pressure [mmHg] | Stress | 333.84 | 1.56 | 1; 27 | 0.222 | 0.05 |
|  | Eplerenone | 0.02 | 0.00 | 1; 27 | 0.993 | <0.01 |
|  | Stress x Eplerenone | 626.41 | 2.93 | 1; 27 | 0.099 | 0.10 |
| Heart Rate (in awake state) [Beats/min] | Stress | 1424.68 | 1.03 | 1; 27 | 0.320 | 0.04 |
|  | Eplerenone | 6804.36 | 4.91 | 1; 27 | **0.035** | 0.15 |
|  | Stress x Eplerenone | 17152.37 | 12.38 | 1; 27 | **0.002** | 0.31 |
| Fractional Shortening [%] | Stress | 1.14 | 0.07 | 1; 27 | 0.794 | <0.01 |
|  | Eplerenone | 0.01 | 0.00 | 1; 27 | 0.982 | <0.01 |
|  | Stress x Eplerenone | 51.19 | 3.13 | 1; 27 | 0.088 | 0.10 |
| Interventricular septum at diastole/body surface area [mm/cm^2^x 10^-3^] | Stress | 0.00 | 44.87 | 1; 27 | **<0.001** | 0.62 |
|  | Eplerenone | 0.00 | 18.37 | 1; 27 | **<0.001** | 0.40 |
|  | Stress x Eplerenone | 0.00 | 0.79 | 1; 27 | 0.383 | 0.03 |
| Left ventricular internal dimension at diastole/body surface area [mm/cm^2^x 10^-3^] | Stress | 0.00 | 4.32 | 1; 27 | **0.047** | 0.14 |
|  | Eplerenone | 0.00 | 4.04 | 1; 27 | 0.055 | 0.13 |
|  | Stress x Eplerenone | 0.00 | 0.23 | 1; 27 | 0.636 | 0.01 |
| Left ventricular posterior wall at diastole/body surface area [mm/cm^2^x 10^-3^] | Stress | 0.00 | 48.66 | 1; 27 | **<0.001** | 0.64 |
|  | Eplerenone | 0.00 | 16.12 | 1; 27 | **<0.001** | 0.37 |
|  | Stress x Eplerenone | 0.00 | 0.72 | 1; 27 | 0.403 | 0.03 |
| Ejection Fraction [%] | Stress | 30.13 | 3.59 | 1; 24 | 0.070 | 0.13 |
|  | Eplerenone | 98.77 | 11.76 | 1; 24 | **0.002** | 0.33 |
|  | Stress x Eplerenone | 1.89 | 0.22 | 1; 24 | 0.640 | 0.01 |
| Left ventricular outflow tract - mean velocity [m/sec] | Stress | 0.01 | 2.07 | 1; 27 | 0.162 | 0.07 |
|  | Eplerenone | 0.04 | 10.41 | 1; 27 | **0.003** | 0.28 |
|  | Stress x Eplerenone | 0.00 | 0.46 | 1; 27 | 0.503 | 0.02 |
| Left ventricular outflow tract - mean pressure gradient [mmHg] | Stress | 0.49 | 4.38 | 1; 27 | **0.046** | 0.14 |
|  | Eplerenone | 1.25 | 11.22 | 1; 27 | **0.002** | 0.29 |
|  | Stress x Eplerenone | 0.02 | 0.21 | 1; 27 | 0.654 | 0.01 |
| Heart Rate (echocardiographic measurement) [Beat/min] | Stress | 3.25 | 0.00 | 1; 27 | 0.947 | <0.01 |
|  | Eplerenone | 137.96 | 0.19 | 1; 27 | 0.664 | 0.01 |
|  | Stress x Eplerenone | 7233.14 | 10.14 | 1; 27 | **0.004** | 0.27 |
| Isovolumic Relaxation Time [ms] | Stress | 18.23 | 2.40 | 1; 26 | 0.134 | 0.08 |
|  | Eplerenone | 0.07 | 0.01 | 1; 26 | 0.923 | <0.01 |
|  | Stress x Eplerenone | 44.36 | 5.84 | 1; 26 | **0.023** | 0.18 |
| e’ (IVS) [m/sec] | Stress | 0.00 | 0.07 | 1; 27 | 0.797 | <0.01 |
|  | Eplerenone | 0.00 | 2.98 | 1; 27 | 0.096 | 0.10 |
|  | Stress x Eplerenone | 0.00 | 0.81 | 1; 27 | 0.377 | 0.03 |
| Mitral E/e’ratio | Stress | 0.08 | 0.02 | 1; 27 | 0.881 | <0.01 |
|  | Eplerenone | 5.29 | 1.58 | 1; 27 | 0.219 | 0.06 |
|  | Stress x Eplerenone | 4.16 | 1.24 | 1; 27 | 0.274 | 0.04 |
| E Vel [m/sec] | Stress | 0.00 | 0.12 | 1; 27 | 0.736 | <0.01 |
|  | Eplerenone | 0.00 | 0.00 | 1; 27 | 0.997 | <0.01 |
|  | Stress x Eplerenone | 0.04 | 9.03 | 1; 27 | **0.006** | 0.25 |
| A Vel [m/sec] | Stress | 0.01 | 2.05 | 1; 27 | 0.164 | 0.07 |
|  | Eplerenone | 0.00 | 0.06 | 1; 27 | 0.806 | <0.01 |
|  | Stress x Eplerenone | 0.00 | 0.65 | 1; 27 | 0.427 | 0.02 |
| Mitral E/A ratio | Stress | 0.17 | 5.17 | 1; 27 | **0.031** | 0.16 |
|  | Eplerenone | 0.00 | 0.03 | 1; 27 | 0.871 | <0.01 |
|  | Stress x Eplerenone | 0.07 | 2.16 | 1; 27 | 0.153 | 0.07 |
| Stroke volume/body surface area [ml/cm^2^] | Stress | 0.00 | 2.37 | 1; 27 | 0.136 | 0.08 |
|  | Eplerenone | 0.00 | 0.17 | 1; 27 | 0.682 | 0.01 |
|  | Stress x Eplerenone | 0.00 | 1.75 | 1; 27 | 0.197 | 0.06 |
| Cardiac output/body surface area [ml/min/cm^2^] | Stress | 0.00 | 0.55 | 1; 27 | 0.463 | 0.02 |
|  | Eplerenone | 0.00 | 1.74 | 1; 27 | 0.198 | 0.06 |
|  | Stress x Eplerenone | 0.00 | 0.66 | 1; 27 | 0.423 | 0.02 |
| Body Weight Change [change from baseline (%)] | Stress | 11277.69 | 162.47 | 1; 27 | **<0.001** | 0.86 |
|  | Eplerenone | 20.76 | 0.30 | 1; 27 | 0.589 | 0.01 |
|  | Stress x Eplerenone | 222.27 | 3.20 | 1; 27 | 0.085 | 0.11 |
| Body surface area [cm^2^] | Stress | 14956.90 | 58.01 | 1; 27 | **<0.001** | 0.68 |
|  | Eplerenone | 9936.55 | 38.54 | 1; 27 | **<0.001** | 0.59 |
|  | Stress x Eplerenone | 811.52 | 3.15 | 1; 27 | 0.087 | 0.10 |
| Corticosterone [mg/ml] | Stress | 766064.66 | 8.04 | 1; 24 | **0.009** | 0.25 |
|  | Eplerenone | 76796.00 | 0.81 | 1; 24 | 0.378 | 0.03 |
|  | Stress x Eplerenone | 284054.17 | 2.98 | 1; 24 | 0.097 | 0.11 |
| Aldosterone [ng/ml] | Stress | 523316.57 | 0.45 | 1; 26 | 0.507 | 0.02 |
|  | Eplerenone | 6103978.13 | 5.29 | 1; 26 | **0.030** | 0.17 |
|  | Stress x Eplerenone | 1091680.83 | 0.95 | 1; 26 | 0.340 | 0.04 |
| Copeptin [pg/ml] | Stress | 12191.36 | 0.68 | 1; 25 | 0.416 | 0.03 |
|  | Eplerenone | 3142.38 | 0.18 | 1; 25 | 0.678 | 0.01 |
|  | Stress x Eplerenone | 4810.96 | 0.27 | 1; 25 | 0.608 | 0.01 |
| Left ventricular mRNA glucocorticoid receptor (LV mRNA GR) ∆Ct | Stress | 10.47 | 2.34 | 1; 27 | 0.138 | 0.08 |
|  | Eplerenone | 15.66 | 3.50 | 1; 27 | 0.072 | 0.11 |
|  | Stress x Eplerenone | 31.21 | 6.98 | 1; 27 | **0.014** | 0.21 |
| Left ventricular mRNA mineralocorticoid receptor (LV mRNA MR) ∆Ct | Stress | 3.50 | 1.04 | 1; 27 | 0.317 | 0.04 |
|  | Eplerenone | 11.99 | 3.56 | 1; 27 | 0.070 | 0.12 |
|  | Stress x Eplerenone | 6.08 | 1.81 | 1; 27 | 0.190 | 0.06 |
| Left ventricular mRNA brain natriuretic peptide ∆Ct | Stress | 1.29 | 0.30 | 1; 27 | 0.590 | 0.01 |
|  | Eplerenone | 10.16 | 2.33 | 1; 27 | 0.138 | 0.08 |
|  | Stress x Eplerenone | 0.29 | 0.07 | 1; 27 | 0.800 | <0.01 |
|  | Eplerenone | 0.00 | 0.68 | 1; 27 | 0.417 | 0.02 |
|  | Stress x Eplerenone | 0.00 | 0.18 | 1; 27 | 0.679 | 0.01 |
| Mean proMMP-2 [activity] | Stress | 0.00 | 0.91 | 1; 21 | 0.352 | 0.04 |
|  | Eplerenone | 0.02 | 3.64 | 1; 21 | 0.070 | 0.15 |
|  | Stress x Eplerenone | 0.00 | 0.07 | 1; 21 | 0.796 | <0.01 |
| Mean MMP-2 activity [activity] | Stress | 0.97 | 32.65 | 1; 27 | **<0.001** | 0.55 |
|  | Eplerenone | 1.25 | 42.18 | 1; 27 | **<0.001** | 0.61 |
|  | Stress x Eplerenone | 0.12 | 3.95 | 1; 27 | 0.057 | 0.13 |
| MMP-9 activity [activity] | Stress | 0.25 | 6.80 | 1; 24 | **0.015** | 0.22 |
|  | Eplerenone | 0.85 | 22.79 | 1; 24 | **<0.001** | 0.49 |
|  | Stress x Eplerenone | 0.01 | 0.34 | 1; 24 | 0.567 | 0.01 |
| Grooming Latency [sec] | Stress | 10250.25 | 2.81 | 1; 27 | 0.105 | 0.09 |
|  | Eplerenone | 21653.26 | 5.93 | 1; 27 | **0.022** | 0.18 |
|  | Stress x Eplerenone | 1692.71 | 0.46 | 1; 27 | 0.502 | 0.02 |
| Grooming Time [sec] | Stress | 10170.01 | 12.85 | 1; 27 | **0.001** | 0.32 |
|  | Eplerenone | 11802.27 | 14.91 | 1; 27 | **0.001** | 0.36 |
|  | Stress x Eplerenone | 13.85 | 0.02 | 1; 27 | 0.896 | <0.01 |

**Table S2.** The results of Tukey post-hoc test

| Variable | Groups | | *p* |
| --- | --- | --- | --- |
| Heart Rate (in awake state) [Beats/min] | C | S | **0.020** |
|  | C | E | 0.802 |
|  | C | SE | 0.837 |
|  | S | E | 0.117 |
|  | S | SE | **0.002** |
|  | E | SE | 0.294 |
| Heart Rate (echocardiographic measurement) [Beat/min] | C | S | 0.158 |
|  | C | E | 0.079 |
|  | C | SE | 0.994 |
|  | S | E | 0.983 |
|  | S | SE | 0.222 |
|  | E | SE | 0.114 |
| Isovolumic Relaxation Time [ms] | C | S | 0.053 |
|  | C | E | 0.375 |
|  | C | SE | 0.736 |
|  | S | E | 0.654 |
|  | S | SE | 0.307 |
|  | E | SE | 0.920 |
| E Vel [m/sec] | C | S | 0.272 |
|  | C | E | 0.183 |
|  | C | SE | 0.995 |
|  | S | E | 0.995 |
|  | S | SE | 0.159 |
|  | E | SE | 0.100 |
| Left ventricular mRNA glucocorticoid receptor (LV mRNA GR) ∆Ct. | C | S | **0.035** |
|  | C | E | 0.950 |
|  | C | SE | 0.995 |
|  | S | E | 0.092 |
|  | S | SE | **0.016** |
|  | E | SE | 0.854 |
