# Supplementary material for: Cardiac response to chronic restraint stress involves mineralocorticoid receptors in male Sprague–Dawley rats
Source: Physiol Rep. 2025 Oct 9;13(19):e70549. doi: 10.14814/phy2.70549 (PMC12510903; doi:10.14814/phy2.70549)
Supplement: Supplementary file 1 — Appendix S1. [file PHY2-13-e70549-s001.zip › Table_S3.docx]

**Table S3.** The effect of stress, eplerenone and interactions of stress and eplerenone on blood pressure, heart rate, grooming behaviour and body weight change.

|  | C | S | SE | E |
| --- | --- | --- | --- | --- |
| SBP [mmHg] | 135.52 ± 18.83 | 155.23 ± 17.22 | 139.5 ± 11.5 | 142.41 ± 15.43 |
| DBP [mmHg] | 92.69 ± 16.06 | 108.27 ± 13.33 | 99.22 ± 14.02 | 101.65 ± 15.17 |
| HR [beats/min] | 306.91 ± 61.96 | 367.61 ± 34.91 | 290.81 ± 17.49 | 324.35 ± 22.99 |
| Grooming Latency [sec] | 90.86 ± 20.31 | 39.63 ± 14.87 | 107.38 ± 60.84 | 129.00 ± 99.0 |
| Grooming Time [sec] | 62.43 ± 18.64 | 97.38 ± 38.25 | 59.63 ± 31.99 | 22.0 ± 16.41 |
| Body weight change [%]^*^ | 28.27 ± 8.08 | -4.58 ± 4.18 | -8.3 ± 9.35 | - 1. ± 10.34 |

Results are presented as mean ± SD. SBP, systolic blood pressure; DBP, diastolic blood pressure; HR, heart rate; C- control group; S- stressed, untreated group; SE- stressed and eplerenone-treated group; E- eplerenone-treated, non-stressed group.

^*^ Percentage change in body weight within the group between day 28 and day 1 of the experiment.
