# Supplementary material for: Cardiac response to chronic restraint stress involves mineralocorticoid receptors in male Sprague–Dawley rats
Source: Physiol Rep. 2025 Oct 9;13(19):e70549. doi: 10.14814/phy2.70549 (PMC12510903; doi:10.14814/phy2.70549)
Supplement: Supplementary file 1 — Appendix S1. [file PHY2-13-e70549-s001.zip › Table_S4.docx]

**Table S4.** The effect of stress, eplerenone and interactions of stress and eplerenone on concentrations of plasma hormones.

|  | C | S | SE | E |
| --- | --- | --- | --- | --- |
| Corticosterone [μg/ml] | 0.805 ± 0.239 | 1.338 ± 0.433 | 1.032 ± 0.322 | 0.902 ± 0.18 |
| Aldosterone [ng/ml] | 1.954 ± 1.774 | 1.307 ± 1.247 | 0.785 ± 0.418 | 0.668 ± 0.281 |
| Copeptin [ng/ml] | 0.147 ± 0.028 | 0.214 ± 0.146 | 0.209 ± 0.169 | 0.194 ± 0.127 |

Results are presented as mean ± SD. C- control group; S- stressed, untreated group; SE- stressed and eplerenone-treated group; E- eplerenone-treated, non-stressed group.
