# Supplementary material for: Cardiac response to chronic restraint stress involves mineralocorticoid receptors in male Sprague–Dawley rats
Source: Physiol Rep. 2025 Oct 9;13(19):e70549. doi: 10.14814/phy2.70549 (PMC12510903; doi:10.14814/phy2.70549)
Supplement: Supplementary file 1 — Appendix S1. [file PHY2-13-e70549-s001.zip › Table_S5.docx]

**Table S5.** The effect of stress, eplerenone and interactions of stress and eplerenone on the expression of MR mRNA, GR mRNA and BNP mRNA in the left ventricle

|  | C | S | SE | E |
| --- | --- | --- | --- | --- |
| GR mRNA [ΔCt] | -0.88 ± 0.97 | -4.05 ± 2.87 | -0.62 ± 2.64 | -1.46 ± 1.12 |
| MR mRNA [ΔCt] | 2.75 ± 1.16 | 1.19 ± 2.43 | 3.32 ± 2.0 | 3.11 ± 1.39 |
| BNP mRNA [ΔCt] | -1.38 ± 1.58 | -1.16 ± 2.5 | 0.17 ± 2.08 | -0.43 ± 2.02 |

Results are presented as mean ± SD. GR mRNA, glucocorticoid receptor mRNA; MR mRNA, mineralocorticoid receptor mRNA; BNP mRNA, brain natriuretic peptide mRNA; C- control group; S- stressed, untreated group; SE- stressed and eplerenone-treated group; E- eplerenone-treated, non-stressed group.
