# Supplementary material for: Cardiac response to chronic restraint stress involves mineralocorticoid receptors in male Sprague–Dawley rats
Source: Physiol Rep. 2025 Oct 9;13(19):e70549. doi: 10.14814/phy2.70549 (PMC12510903; doi:10.14814/phy2.70549)
Supplement: Supplementary file 1 — Appendix S1. [file PHY2-13-e70549-s001.zip › Table_S6.docx]

**Table S6.** The effect of stress, eplerenone and interactions of stress and eplerenone on matrix metalloproteinases’ activity in the left ventricle.

|  | C | S | SE | E |
| --- | --- | --- | --- | --- |
| MMP-2 | 0.49 ± 0.12 | 0.97 ± 0.26 | 0.44 ± 0.17 | 0.21 ± 0.07 |
| proMMP-2 | 0.13 ± 0.05 | 0.16 ± 0.11 | 0.1 ± 0.07 | 0.08 ± 0.02 |
| MMP-9 | 0.46 ± 0.32 | 0.61 ± 0.27 | 0.3 ± 0.05 | 0.06 ± 0.03 |

Results are presented as mean ± SD. MMP-2, matrix metalloproteinase-2; proMMP-2, pro-matrix metalloproteinase-2; MMP-9, matrix metalloproteinase-9; C- control group; S- stressed, untreated group; SE- stressed and eplerenone-treated group; E- eplerenone-treated, non-stressed group.
