# Supplementary material for: Cardiac response to chronic restraint stress involves mineralocorticoid receptors in male Sprague–Dawley rats
Source: Physiol Rep. 2025 Oct 9;13(19):e70549. doi: 10.14814/phy2.70549 (PMC12510903; doi:10.14814/phy2.70549)
Supplement: Supplementary file 1 — Appendix S1. [file PHY2-13-e70549-s001.zip › Table_S7.docx]

**Table S7.** The effect of stress, eplerenone and interactions of stress and eplerenone on echocardiographic parameters

|  | C | S | SE | E |
| --- | --- | --- | --- | --- |
| IVSd/BSA [mm/cm^2^ x 10^-3^] | 0.0032 ± 0.0002 | 0.0035 ± 0.0002 | 0.0034 ± 0.0001 | 0.003 ± 0.0001 |
| LVIDd/BSA [mm/cm^2^ x 10^-3^] | 0.0172 ± 0.0008 | 0.0179 ± 0.0012 | 0.0173 ± 0.0012 | 0.0163 ± 0.001 |
| LVPWd/BSA [mm/cm^2^ x 10^-3^] | 0.0032 ± 0.0002 | 0.0035 ± 0.0002 | 0.0034 ± 0.0001 | 0.0029 ± 0.0001 |
| EF [%] | 75.88 ± 2.64 | 77.44 ± 2.99 | 74.19 ± 3.34 | 71.58 ± 2.62 |
| FS [%] | 42.14 ± 4.02 | 44.33 ± 3.58 | 41.79 ± 3.75 | 44.75 ± 4.72 |
| SV/BSA [ml/cm^2^] | 0.00022 ± 0.00006 | 0.00022 ± 0.00005 | 0.00023 ± 0.00002 | 0.00019 ± 0.00003 |
| CO/BSA [ml/min/cm^2^] | 0.17 ± 0.04 | 0.17 ± 0.03 | 0.18 ± 0.03 | 0.19 ± 0.02 |
| E/A ratio | 1.76 ± 0.17 | 1.71 ± 0.21 | 1.6 ± 0.14 | 1.85 ± 0.21 |
| E/e’ | 13.57 ± 1.68 | 14.4 ± 1.13 | 12.84 ± 1.93 | 13.48 ± 2.34 |
| IVRT [ms] | 21.71 ± 2.03 | 17.71 ± 3.69 | 20.25 ± 2.74 | 19.38 ± 2.35 |

Results are presented as mean ± SD. IVSd, interventricular septum at diastole; LVIDd left ventricular internal dimension at diastole; LVOT, left ventricular outflow tract; LVPWd, left ventricular posterior wall at diastole; EF, ejection fraction; FS, fractional shortening; SV, stroke volume; CO, cardiac output; E/A, early to late diastolic transmitral flow velocity; E/e’, early diastolic transmitral flow velocity to early diastolic mitral annular velocity; IVRT, isovolumic relaxation time; C- control group; S- stressed, untreated group; SE- stressed and eplerenone-treated group; E- eplerenone-treated, non-stressed group.
